# Effects of short-term hyperoxia on erythropoietin levels and microcirculation in critically Ill patients: a prospective observational pilot study

**DOI:** 10.1186/s12871-017-0342-2

**Published:** 2017-03-23

**Authors:** Abele Donati, Elisa Damiani, Samuele Zuccari, Roberta Domizi, Claudia Scorcella, Massimo Girardis, Alessia Giulietti, Arianna Vignini, Erica Adrario, Rocco Romano, Laura Mazzanti, Paolo Pelaia, Mervyn Singer

**Affiliations:** 1 Anesthesia and Intensive Care Unit, Department of Biomedical Sciences and Public Health, Università Politecnica delle Marche, via Tronto 10, 6126 Torrette di Ancona, Italy; 2 Department of Anesthesiology and Intensive Care, Modena University Hospital, L.go del Pozzo 71, 41100 Modena, Italy; 3 Department of Clinical Sciences, Section of Biochemistry, Università Politecnica delle Marche, via Tronto 10, 60126 Torrette di Ancona, Italy; 4 Bloomsbury Institute of Intensive Care Medicine, University College London, Gower Street, London, WC1E 6BT UK

**Keywords:** Erythropoietin, Anemia, Normobaric hyperoxia, Microcirculation

## Abstract

**Background:**

The normobaric oxygen paradox states that a short exposure to normobaric hyperoxia followed by rapid return to normoxia creates a condition of ‘relative hypoxia’ which stimulates erythropoietin (EPO) production. Alterations in glutathione and reactive oxygen species (ROS) may be involved in this process. We tested the effects of short-term hyperoxia on EPO levels and the microcirculation in critically ill patients.

**Methods:**

In this prospective, observational study, 20 hemodynamically stable, mechanically ventilated patients with inspired oxygen concentration (FiO_2_) ≤0.5 and PaO_2_/FiO_2_ ≥ 200 mmHg underwent a 2-hour exposure to hyperoxia (FiO_2_ 1.0). A further 20 patients acted as controls. Serum EPO was measured at baseline, 24 h and 48 h. Serum glutathione (antioxidant) and ROS levels were assessed at baseline (t0), after 2 h of hyperoxia (t1) and 2 h after returning to their baseline FiO_2_ (t2). The microvascular response to hyperoxia was assessed using sublingual sidestream dark field videomicroscopy and thenar near-infrared spectroscopy with a vascular occlusion test.

**Results:**

EPO increased within 48 h in patients exposed to hyperoxia from 16.1 [7.4–20.2] to 22.9 [14.1–37.2] IU/L (*p* = 0.022). Serum ROS transiently increased at t1, and glutathione increased at t2. Early reductions in microvascular density and perfusion were seen during hyperoxia (perfused small vessel density: 85% [95% confidence interval 79–90] of baseline). The response after 2 h of hyperoxia exposure was heterogeneous. Microvascular perfusion/density normalized upon returning to baseline FiO_2_.

**Conclusions:**

A two-hour exposure to hyperoxia in critically ill patients was associated with a slight increase in EPO levels within 48 h. Adequately controlled studies are needed to confirm the effect of short-term hyperoxia on erythropoiesis.

**Trial registration:**

ClinicalTrials.gov (www.clinicaltrials.gov), NCT02481843, registered 15th June 2015, retrospectively registered

## Background

Anemia is a frequent problem in critically ill intensive care patients [1]. Inhibition of erythropoietin (EPO) production induced by inflammatory cytokines contributes to the etiology of critical illness anemia [2–4]. A diminished oxygen (O_2_) content due to anemia or hypoxaemia is the physiologic stimulus for EPO synthesis [5]. Reports suggest that a sudden decrease in tissue O_2_ levels after a short-term exposure to normobaric hyperoxia can stimulate EPO synthesis by producing a state of “relative” hypoxia (normobaric oxygen paradox, NOP) [6, 7]. However, studies are needed to demonstrate the effectiveness of this procedure, to define the optimal dose and frequency of exposure [8], and to confirm the safety of transient exposure to hyperoxia in ICU patients. Hyperoxia induces oxidative damage to various organs [9], coronary and systemic vasoconstriction [10] with a fall in cardiac output [11], and a possible decrease in regional O_2_ delivery [12]. Exposure to arterial hyperoxia is also associated with higher mortality in certain ICU patient subsets [13].

We aimed to test the effectiveness of the NOP on increasing serum EPO levels in critically ill patients. Secondly, we explored the safety of hyperoxia exposure by evaluating the microcirculatory response to hyperoxia and the effects upon circulating levels of nitric oxide (NO), reactive O_2_ species (ROS) and the antioxidant, glutathione (GSH).

## Methods

This prospective, non-randomized study was approved by the Institutional Review Board of the Azienda Ospedaliera Universitaria Ospedali Riuniti of Ancona, Italy (Protocol nr. 212638; NCT02481843 www.clinicaltrials.gov). Written informed consent was obtained from the enrolled patients or their next of kin. Adult (≥18-year old) patients admitted to the 12-bed medical-surgical ICU of Azienda Ospedaliera Universitaria Ospedali Riuniti of Ancona, Italy between April 2013 and March 2015 and requiring mechanical ventilation with an inspired O_2_ fraction (FiO_2_) ≤0.5 and PaO_2_/FiO_2_ ≥ 200 mmHg were eligible to participate. Exclusion criteria were: hemoglobin (Hb) <9 g/dL, acute bleeding or blood transfusion during the study period; surgical interventions during the study period; acute or chronic renal failure; hemodynamic instability; chronic obstructive pulmonary disease; pregnancy; factors impeding evaluation of the sublingual microcirculation (oral surgery or maxillo-facial trauma). In all patients, blood pressure was monitored with an arterial catheter. Sedation and analgesia, fluids and vasopressors were provided according to individual needs.

### Interventions

Forty patients were enrolled in total and were allocated in the hyperoxia or control group using a before-after method. The first 20 patients (hyperoxia group) underwent a 2-hour period of normobaric hyperoxia (FiO_2_ 1.0), according to the protocol applied by Balestra et al. [6]. No variation in FiO_2_ was applied to the last 20 patients (control group). All patients were enrolled in the morning and hyperoxia was performed between 10.00 and 14.00 h to minimize variability due to the circadian rhythm of EPO production. No variations to sedation or vasopressor dosing or ventilator settings were applied during the study period.

### Measurements

On the study day, measurements were taken at baseline (t0), after 2 h of breathing 1.0 FiO_2_ (hyperoxia, t1) and 2 h after returning to baseline FiO_2_ (t2). Measures included body temperature, heart rate (HR), mean arterial pressure (MAP), arterial and central venous blood gases, arterial lactate, evaluation of the sublingual microcirculation and peripheral microvascular (StO_2_) oxygenation. The same measures were performed in the control group but with no period of hyperoxia. Serum EPO, reticulocyte count, Hb and hematocrit were measured at 8 am in all patients on the study day, and at 24 and 48 h.

### Microcirculation assessment

The sublingual microcirculation was evaluated at five different sites using sidestream dark field (SDF) videomicroscopy (Microscan, Microvision Medical, Amsterdam, NL), as previously described [14–17]. Three images per time-point were analyzed using the Automated Vascular Analysis software (Microvision Medical BV). Total vessel density (TVD), perfused vessel density (PVD), De Backer score, proportion of perfused vessels (PPV), microcirculatory flow index (MFI), flow heterogeneity index (FHI) and blood flow velocity (BFV) were calculated in small or medium vessels (diameter ≤ or >20 μm, respectively) [16, 18]. Continuous video recording was also performed for ≥2 min on one site during the change in FiO_2_ (either start or end of hyperoxia) to evaluate the early microcirculatory response. Video clips of 10 s’ duration (two per time point) corresponding to before (baseline or 2 h FiO_2_ 1.0) and after (2 min FiO_2_ 1.0 or 2 min after returning to baseline FiO_2_) the change in FiO_2_ were selected and subsequently analyzed.

Near-infrared spectroscopy (NIRS) (InSpectra™ Model 650; Hutchinson Technology Inc., Hutchinson, MN, USA) with a 15 mm-sized probe was used to measure microvascular oxygen saturation (StO_2_) and tissue Hb index (THI) [19] on the thenar eminence at baseline and during a vascular occlusion test [16, 20]. The StO_2_ downslope (%/minute) was calculated as an index of O_2_ consumption, while the StO_2_ upslope (%/minute) and the area under the curve (AUC) of the hyperemic response were obtained to assess microvascular reactivity [20].

### Immunoassays

In the first 12 patients in each group, arterial blood samples (10 mL) were taken at baseline, t1 and t2 and immediately centrifuged; plasma and serum were stored at −70 °C. A marker of NO production was measured as plasma nitrite/nitrate using a Nitric Oxide Colorimetric Detection Kit (Cat. No. K023-H1, Arbor Assays, Ann Arbor, MI, USA) while GSH levels were measured using a Glutathione Colorimetric Detection Kit (Cat. No. K006-H1 - Arbor Assays) following the manufacturer’s instructions. High Reactive Oxygen Species (hROS) levels were determined by hydroxyphenyl fluorescein (Cell Technology Inc, Mountain View, CA, USA), which selectively detects hydroxyl radical (●OH) and peroxynitrite (ONOO^−^) [21]. Immunoassays could not be performed in all patients for economic reasons.

### Statistical analysis

This was performed using GraphPad Prism Version 6 (GraphPad Software, La Jolla, CA, USA). Normality of distribution was checked using the Kolmogorov-Smirnov test. The data were expressed as mean ± standard deviation (SD) for normally distributed variables or median [1st-3rd quartiles] for non-normally distributed variables. One-way analysis of variance (ANOVA) for repeated measures with Bonferroni post-hoc testing (or Friedman’s test with Dunn’s multiple comparison test for non-normally distributed variables) was used to evaluate changes over time in the same group. For normally distributed variables, the two-way ANOVA for repeated measures with Bonferroni post-hoc testing was used to evaluate differences between the groups. For non-normally distributed variables, the Mann-Whitney *U* test was applied to evaluate differences between the two groups at the same time-point. Spearman correlation coefficient was calculated to assess correlations between variables. The alpha level of significance was set *a priori* at 0.05.

## Results

A total of 791 patients was screened during the 2-year study period. Of these, 617 patients were excluded because they did not meet the predefined inclusion criteria or had exclusion criteria; 105 patients were excluded as already enrolled in another study; 24 patients denied consent to participation in the study; 45 patients were not enrolled due to the unavailability of the investigators or SDF/NIRS devices. Median age was 74 [53–81] years in the hyperoxia group and 65 [56–73] years in the control group. The male:female ratio was 10:10 and 14:6 in the hyperoxia and control groups, respectively. Admission diagnoses were neurological (hyperoxia group: 4 patients, controls: 12), polytrauma (hyperoxia: 5, controls: 7), post-cardiac arrest (hyperoxia: 7, controls: 1) and sepsis (hyperoxia: 3, none in the control group). The Sequential Organ Failure Assessment (SOFA) score was 8.5 [6.2–10] and 8 [6–9] in the hyperoxia and control groups, respectively.

### Hemodynamic and blood gas data

The PaO_2_ increased to >400 mmHg during hyperoxia. A significant decrease in MAP (*p* < 0.01 versus t1) and increase in HR (*p* < 0.05 versus t0 and t1) were found at t2 in the hyperoxia group. Central venous O_2_ saturation (ScvO_2_) increased in the hyperoxia group at t1 (*p* < 0.001 versus t0 and t2). Arterial lactate levels increased in the hyperoxia group and were higher than those seen in the control group at t1 and t2 (Table 1).

**Table 1:** Variations in hemodynamics and blood gas parameters under hyperoxia and after the return to baseline FiO_2_

	t0	t1	t2	p (time)^a^	p (interaction)^b^
Heart Rate (bpm)					0.813
*Hyperoxia*	70 ± 20	71 ± 22	77 ± 22^##‡^	0.008	
*Controls*	67 ± 19	66 ± 19	72 ± 25	0.086	
Mean Arterial Pressure (mmHg)					-
*Hyperoxia*	86 [70–91]	87 [81–97]	83 [72–87]*	0.008	
*Controls*	87 [79–101]	88 [84–97]	85 [81–92]	0.462	
Body temperature (°C)					0.176
*Hyperoxia*	36.1 ± 1.1	36.3 ± 0.9	36.3 ± 1.1*	0.407	
*Controls*	36.7 ± 0.8	36.7 ± 0.9	37.1 ± 0.8^#‡^	0.010	
PaO_2_ (mmHg)					<0.001
*Hyperoxia*	108 ± 35	433 ± 109^###^***	111 ± 26^‡‡‡^	<0.001	
*Controls*	118 ± 33	119 ± 28	115 ± 33	0.622	
SaO_2_ (%)					-
*Hyperoxia*	99.7 [98.9–100]	100[100–100]^###^***	99.8 [99.1–100]^‡‡^	<0.001	
*Controls*	100 [99.6–100]	99.9 [99.5–100]	99.8 [99.5–100]	0.863	
PaO_2_/FiO_2_ (mmHg)					<0.001
*Hyperoxia*	290 ± 93	435 ± 111^###^***	287 ± 82^‡‡‡^	<0.001	
*Controls*	314 ± 108	315 ± 93	303 ± 99	0.330	
ScvO_2_ (%)					-
*Hyperoxia*	79 [70–83]	86 [74–90]^###^	76 [72–81]^‡‡‡^	<0.001	
*Controls*	82 [74–84]	80 [75–85]	79 [75–84]	0.838	
pH					0.696
*Hyperoxia*	7.48 ± 0.07	7.49 ± 0.07	7.48 ± 0.07	0.516	
*Controls*	7.45 ± 0.06	7.45 ± 0.05	7.45 ± 0.06	0.966	
PaCO_2_ (mmHg)					-
*Hyperoxia*	38 [36–42]	39 [34–42]	39 [36–42]	0.826	
*Controls*	38 [34–42]	39 [35–43]	41 [35–42]	0.145	
Base excess (mmol/L)					0.971
*Hyperoxia*	5.0 ± 5.2	5.3 ± 5.3	5.6 ± 4.9	0.430	
*Controls*	2.9 ± 3.8	3.1 ± 4.1	3.3 ± 3.6	0.288	
Lactate (mmol/L)					-
*Hyperoxia*	1.1 [0.7–1.5]	1.2 [1.1–2.0]**	1.3 [1.0–1.8]*	0.037	
*Controls*	0.9 [0.7–1.2]	0.9 [0.7–1.2]	0.9 [0.7–1.2]	0.616	

Data are expressed as mean ± standard deviation or median [1st-3rd quartile], as appropriate (t0): baseline; (t1): 2 h 1.0 FiO2; (t2): 2 h after the return to baseline FiO_2_

^a^ Overall effect of time on the variance in each group. One-way ANOVA for repeated measures with Bonferroni’s post hoc test or Friedman test with Dunn’s test for multiple comparisons, as appropriate

^b^ Test of interaction between time and groups. Two-way ANOVA for repeated measures. This was applicable only to normally distributed variables

*p < 0.05, **p < 0.01, ***p < 0.001 versus controls (same time point); two-way ANOVA for repeated measures with Bonferroni post-hoc test or Mann-Whitney test, as appropriate

^#^ *p* < 0.05, ^##^ *p* < 0.01, ^###^ *p* < 0.001 versus t0; ^‡^ *p* < 0.05, ^‡‡^ *p* < 0.01, ^‡‡‡^ *p* < 0.001 versus t1; One-way ANOVA for repeated measures with Bonferroni’s post hoc test or Friedman test with Dunn’s test for multiple comparisons, as appropriate

### Effects on erythropoiesis

EPO levels rose in the hyperoxia group (*p* < 0.05) and were significantly higher at 48 h compared to baseline (*p* < 0.05). No changes were seen in the control group. The reticulocyte count and Hb levels fell over time in both groups (Table 2).

**Table 2:** Variations in erythropoietin, reticulocyte count, haemoglobin and haematocrit in the two groups

	Baseline	24 h	48 h	p (time)^a^	p (interaction)^b^
Erythropoietin (IU/L)					-
*Hyperoxia*	16.1 [7.4–20.2]	20.1 [10.7–31.2]	22.9 [14.1–37.2]^#^	0.022	
*Controls*	12.8 [7.3–22.2]	14.9 [9.2–18.6]	14.8 [7.9–24.8]	0.692	
Reticulocytes (*10^3^/mm^3^)					-
*Hyperoxia*	55.9 [39.4–67.4]	47.3 [33.1–62.2]^##^	46.2 [29.8–66.2]	0.010	
*Controls*	60.2 [46.3–78.7]	52.5 [42.9–65.9]	50.7 [44.1–64.5]^#^	0.027	
Hemoglobin (g/dL)					0.601
*Hyperoxia*	10.7 ± 1.4	10.2 ± 1.3	10.1 ± 1.3^#^	0.013	
*Controls*	11.7 ± 2.0	11.3 ± 1.8	11.3 ± 1.7	0.027	
Hematocrit (%)					0.053
*Hyperoxia*	32 ± 6	31 ± 6	30 ± 6^#^*	0.029	
*Controls*	34 ± 8	34 ± 8	34 ± 8	0.675	

Data are expressed as mean ± standard deviation or median [1st-3rd quartile], as appropriate

^a^ Overall effect of time on the variance in each group. One-way ANOVA for repeated measures with Bonferroni’s post hoc test or Friedman test with Dunn’s test for multiple comparisons, as appropriate

^b^ Test of interaction between time and groups. Two-way ANOVA for repeated measures. This was applicable only to normally distributed variables

*p < 0.05 versus controls (same time point); two-way ANOVA for repeated measures with Bonferroni post-hoc test

### Effects on the sublingual microcirculation

**Fig. 1:**
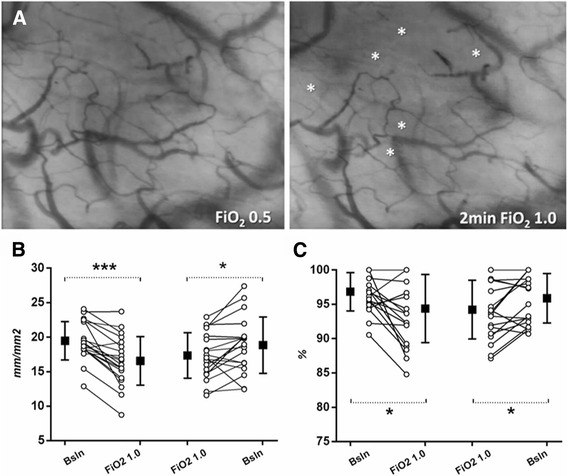
Early microcirculatory response to acute variations in the FiO_2_. **a** *:* Sidestream Dark Field imaging of the same site of sublingual microcirculation in one patient before (FiO_2_ 0.5) and after 2 min of exposure to hyperoxia (2 min FiO_2_ 1.0). Stars indicate regions of microvascular de-recruitment. **b** and **c** *:* Individual and mean (SD) changes in perfused vessel density (**b**) and proportion of perfused vessels (**c**) during acute variations in the FiO_2_ in the hyperoxia group (*n* = 20). The same region of the sublingual mucosa was assessed continuously at each time point (start hyperoxia and end hyperoxia) for at least 2 min . *p < 0.05, ***p < 0.001, paired *t*-test

Continuous microcirculatory assessment of the same site during changes in the FiO_2_ showed that the first 2 min of hyperoxia were associated with an early and consistent reduction in TVD (87% [95% confidence interval (CI) 82–91] of baseline for small vessels), PVD (85% [79–90] of baseline for small vessels) and PPV (97% [96–99] of baseline for small vessels) (Fig. 1). When compared to measures taken after breathing an FiO_2_ of 1.0 for 2 h, these parameters increased on return to the baseline FiO_2_ (TVD: 107% [100–114], PVD: 109% [102–117], PPV: 102% [100–103] (Fig. 1).

Evaluation of the microcirculation at 2-hour intervals showed a more heterogeneous response. A trend was seen towards a reduction in the De Backer score, TVD and PVD for small vessels at t1, with normalization at t2 (Table 3). The variation in TVD after 2 h hyperoxia (t1-t0) was inversely correlated with the change seen 2 h after return to the baseline FiO_2_ (t2-t1) (*r* = −0.52 [95% CI −0.78, −0.10]). The microcirculation remained stable over time in the control group, which showed a significantly higher De Backer score, TVD and PVD for small and medium vessels as compared to the hyperoxia group at all time-points (Table 3).

**Table 3:** Sublingual and peripheral microvascular changes after a 2-hour exposure to hyperoxia and 2 h after return to baseline FiO_2_

	t0	t1	t2	p (time)^a^	p (interaction)^b^
TVD small (mm/mm^2^)					-
*Hyperoxia*	18.5 [16.5–21.6]**	16.4 [14.6–21.6]**	19.3 [18.2–21.3]^‡^*	0.035	
*Controls*	21.7 [20.0–23.8]	22.2 [18.8–24.3]	21.0 [19.8–23.7]	0.705	
TVD medium (mm/mm^2^)					-
*Hyperoxia*	0.9 [0.4–1.6]	0.5 [0.4–0.9]**	0.7 [0.3–1.3]	0.268	
*Controls*	1.2 [0.8–1.8]	1.0 [0.7–1.8]	1.1 [0.6–1.7]	0.861	
PVD small (mm/mm^2^)					-
*Hyperoxia*	17.4 [16.3–20.8]**	15.4 [13.2–20.3]**	18.4 [17.1–19.9]*	0.058	
*Controls*	20.1 [18.9–22.6]	21.0 [18.0–23.3]	20.0 [18.5–22.9]	0.549	
PVD medium (mm/mm^2^)					0.178
*Hyperoxia*	0.9 ± 0.6	0.6 ± 0.5*	0.8 ± 0.6	0.142	
*Controls*	1.2 ± 0.6	1.2 ± 0.6	1.1 ± 0.7	0.851	
De Backer score (n/mm)					-
*Hyperoxia*	11.2 [9.9–13.2]**	9.7 [8.8–13.2]**	11.8 [10.0–13.4]^‡^**	0.019	
*Controls*	14.5 [12.0–15.8]	14.1 [11.6–15.8]	14.1 [12.7–16]	0.951	
MFI small (AU)					-
*Hyperoxia*	2.74 [2.50–2.90]	2.67 [2.50–2.90]	2.75 [2.52–2.92]	0.713	
*Controls*	2.67 [2.44–2.90]	2.75 [2.60–3.00]	2.79 [2.67–2.98]	0.099	
PPV small (%)					0.223
*Hyperoxia*	96 ± 3	94 ± 4	95 ± 3	0.149	
*Controls*	94 ± 4	95 ± 3	95 ± 4	0.396	
FHI small (AU)					-
*Hyperoxia*	0.14 [0.08–0.21]	0.18 [0.09–0.30]*	0.10 [0.02–0.20]	0.551	
*Controls*	0.09 [0.02–0.19]	0.09 [0.00–0.18]	0.09 [0.00–0.18]	0.290	
BFV (μm/s)					0.631
*Hyperoxia*	497 ± 63	489 ± 81	502 ± 45	0.741	
*Controls*	520 ± 56	519 ± 54	531 ± 65	0.537	
StO_2_ (%)					0.655
*Hyperoxia*	80 ± 8	82 ± 8	82 ± 7	0.261	
*Controls*	81 ± 9	81 ± 8	82 ± 9	0.824	
StO_2_ downslope (%/min)					-
*Hyperoxia*	−7.9 [−9.1, −7.0]	−7.7 [−9.3, −6.8]	−9.4 [−11.0, −7.9]^‡^	0.003	
*Controls*	−8.1 [−9.7, −6.8]	−7.9 [−10.1, −7.0]	−8.6 [−9.9, −6.9]	0.212	
StO_2_ upslope (%/min)					-
*Hyperoxia*	133 [98–243]	194 [145–274]	192 [140–244]	0.101	
*Controls*	194 [150–228]	193 [139–262]	219 [132–263]	0.247	
StO_2_ area under the curve (%*min)					0.284
*Hyperoxia*	14.6 ± 8.8	18.0 ± 11.8	13.2 ± 7.6	0.066	
*Controls*	20.3 ± 15.1	19.8 ± 14.7	18.3 ± 13.3	0.660	
THI (AU)					0.884
*Hyperoxia*	11.0 ± 3.2	11.4 ± 3.8	11.8 ± 3.2	0.374	
*Controls*	12.6 ± 2.2	12.7 ± 3.5	13.0 ± 3.1	0.801	

Data are expressed as mean ± standard deviation or median [1st-3rd quartile], as appropriate. (t0): baseline; (t1): 2 h 1.0 FiO2; (t2): 2 h after the return to baseline FiO_2_. *TVD* total vessel density, *PVD* perfused vessel density, *PPV* proportion of perfused vessels, *MFI* microvascular flow index, *FHI* flow heterogeneity index, *BFV* blood flow velocity

^a^ Overall effect of time on the variance in each group. One-way ANOVA for repeated measures with Bonferroni’s post hoc test or Friedman test with Dunn’s test for multiple comparisons, as appropriate

^b^ Test of interaction between time and groups. Two-way ANOVA for repeated measures. This was applicable only to normally distributed variables

*p < 0.05, **p < 0.01 versus controls (same time point); two-way ANOVA for repeated measures with Bonferroni post-hoc test or Mann-Whitney test, as appropriate

^‡^ *p* < 0.05 versus t1; Friedman test with Dunn’s test for multiple comparisons, as appropriate

### NIRS variables

The StO_2_ value was not affected by changes in FiO_2_. The gradient of the StO_2_ downslope was however significantly increased at t2 in the hyperoxia group (*p* < 0.01 versus t1) implying increased O_2_ extraction rate, although no significant difference was found with the control group. The StO_2_ upslope, area under the curve and THI did not show any significant change over time in the hyperoxia group compared to controls. All NIRS parameters remained stable in the control group and no differences were found between the two groups at any time-point (Table 3).

### NO, ROS and GSH

**Fig. 2:**
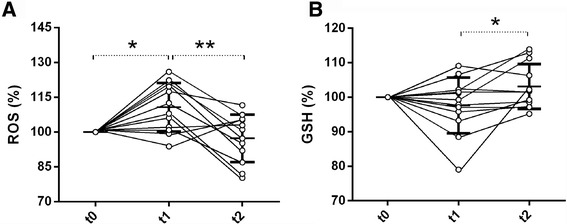
Individual and mean (SD) changes in serum ROS (**a**) and GSH (**b**) levels in the hyperoxia group (*n* = 12), expressed as percentage of baseline values. *p < 0.05, **p < 0.01, one-way ANOVA for repeated measures with Bonferroni’s post hoc test

The percentage variations of ROS and GSH in the hyperoxia group are shown in Fig. 2. ROS increased to 111% [95% CI 104–117] of baseline at t1 (*p* < 0.05 versus t0), and decreased to 97% [91–104] of baseline at t2 (*p* < 0.01 versus t1). GSH did not change at t1 (98% [92–103] of baseline) but increased to 103% [99–107] of baseline at t2 (*p* < 0.05 versus t1). NO did not change significantly over time in either group (Table 4). No correlation was found between changes in nitrite/nitrate and changes in sublingual microvascular parameters.

**Table 4:** Changes in nitrite/nitrate, GSH and ROS after a 2-hour exposure to hyperoxia and 2 h after return to baseline FiO_2_

	t0	t1	t2	p (time)^a^	p (interaction)^b^
Nitrite/nitrate (μM)					0.378
*Hyperoxia (n = 12)*	41 ± 17	45 ± 21	43 ± 18	0.301	
*Controls (n = 12)*	51 ± 18	55 ± 16	59 ± 14	0.198	
GSH (μM)					-
*Hyperoxia (n = 12)*	2.21 [2.12–2.28]	2.17 [2.09–2.25]	2.24 [2.20–2.39]	0.114	
*Controls (n = 12)*	2.19 [2.12–2.33]	2.18 [2.15–2.23]	2.23 [2.17–2.28]	0.273	
ROS (RFU)					-
*Hyperoxia (n = 12)*	2915 [1196–6537]	3109 [1348–6961]	3061 [1261–5841]	0.046	
*Controls (n = 12)*	1773 [1070–3018]	2024 [1135–3069]	1841 [1066–3058]	0.338	

These analyses were restricted to the 12 patients per group. Data are expressed as mean ± standard deviation or median [1st-3rd quartile], as appropriate. (t0): baseline; (t1): 2 h 1.0 FiO2; (t2): 2 h after return to baseline FiO_2_. *RFU* relative fluorescence units

^a^ Overall effect of time on the variance in each group. One-way ANOVA for repeated measures with Bonferroni’s post hoc test or Friedman test with Dunn’s test for multiple comparisons, as appropriate

^b^ Test of interaction between time and groups. Two-way ANOVA for repeated measures. This was applicable only to normally distributed variables

## Discussion

In this prospective observational study on 40 critically ill patients within different disease categories, a single 2-hour exposure to normobaric hyperoxia was associated with a slight increase in serum EPO levels over the following 48 h, despite wide inter-individual variability in the response observed. An initial decrease in microvascular perfusion was seen during hyperoxia, followed by normalization on returning to the baseline FiO_2_. Serum ROS levels were elevated after hyperoxia, while the return to the baseline FiO_2_ was associated with an increase in GSH and normalization of ROS levels.

Intermittent hyperoxia can stimulate erythropoiesis in healthy volunteers and in various patient categories [6, 7, 22, 23], although other reports have questioned the existence of a NOP [24]. Mechanisms underlying the NOP may involve regulation of Hypoxia-Inducible Factor 1-alpha (HIF-1α) expression. During normoxia, HIF-1α undergoes ubiquitination by the Von Hippel Lindau tumor suppressor and degradation into the proteasome. This process is mediated by the oxidized form of glutathione (GSSG). Under hypoxia, the GSH-GSSG ratio increases, thus limiting the inactivation of HIF-1α, which can induce expression of the EPO gene. The increase in ROS during hyperoxia induces *de novo* synthesis of GSH. When returning to normoxia, the increased production of GSH, together with the reduction of GSSG to GSH, creates a “surplus” of GSH that enhances the inactivation of HIF-1α [6]. Our findings are consistent with this theory: serum ROS levels increased after 2 h of hyperoxia, while GSH levels were elevated 2 h after returning to the baseline FiO_2_. This variation in GSH levels may be able to modulate the activity of HIF-1α and EPO production.

Several factors may account for the inter-individual variability observed in the NOP response. Arterial hyperoxia (PaO_2_ > 100 mmHg) was present at baseline in 60% of patients in the hyperoxia group; this may have had either a positive (enhanced induction of GSH synthesis) or negative (higher ROS levels) impact on the EPO response. The two groups were also unbalanced for admission diagnosis so definitive conclusions cannot be drawn. Importantly, baseline Hb levels tended to be lower in the hyperoxia group: this could in itself act as a stimulus for EPO synthesis independent of exposure to hyperoxia.

As EPO secretion normally follows an individual circadian rhythm of production, individual matching of the circadian rhythm is needed to detect an increase in EPO levels at a specific time of the day [8]. This could be particularly important in ICU patients where the normal circadian rhythm of hormone production is frequently abolished due to their underlying illness as well as sedation and disturbances in the sleep-wake cycle [25]. We measured EPO levels at 08.00 h each morning in all patients to limit the impact of this source of variability.

The ideal “dose” of O_2_ for triggering the NOP is poorly described. An excessive increase in ROS (such as that induced by hyperbaric hyperoxia) may suppress rather than stimulate EPO production [6]. An FiO_2_ of 1.0 may not represent optimal dosing for all patients and could have produced varying effects. Repeated rather than single exposures may perhaps be more effective [8].

Arterial hyperoxia causes vasoconstriction, thus reducing tissue perfusion [11, 26]. This cannot be part of an evolutionary innate response as exposure to high concentrations of inspired oxygen can only be traced back to the relatively modern era. Arterial hyperoxia must therefore represent a purely iatrogenic ‘insult’; the ensuing physiological response (vasoconstriction) to the resulting tissue hyperoxia may reproduce adaptive efforts to match O_2_ supply to reduced cellular metabolic needs seen in pathological states such as sepsis where tissue oxygen tensions rise [27]. The corollary of reducing oxygen supply in conditions of relative tissue oxygen excess is a blunting of the increase in ROS production, thereby attenuating toxicity.

Mechanisms underlying this response may involve a down-regulation of the adenosine pathway [28] or modulation of the ROS-mediated prostaglandin and NO pathways [29] with reduced NO bioavailability [30]. In our study, hyperoxia led to an early decrease in microcirculatory vessel density and perfusion after 2 min of exposure. These alterations persisted after 2 h in some patients, while others showed similar or even increased vessel densities as compared with baseline levels. No reduction in plasma NO levels was seen after 2 h hyperoxia, which may reflect the variability observed in microvascular perfusion. An undetected short-lived derangement in the NO pathway may at least partly explain the early microcirculatory alterations observed. Our results suggest that the vasoconstrictor response was more pronounced during an acute increase in arterial PaO_2_ rather than after a more sustained exposure. Of note, continuous monitoring of the microcirculation in the same region of sublingual mucosa is likely to be more sensitive in detecting even minute variations in vessel density compared to intermittent measurements, as it excludes any variability related to the random selection of different areas, albeit averaged over three samples [31, 32].

Microvascular density and perfusion normalized after the return to the baseline FiO_2_. This was associated with a decrease in MAP and an increase in HR after 2 h , suggesting a decrease in systemic vascular resistance. The increased gradient in the StO_2_ downslope at 2 h after returning to baseline FiO_2_ indicates a higher regional O_2_ extraction rate. This likely reflects an increase in local perfusion and O_2_ consumption in this phase. Notably, the microvascular response at 2 h after return to normoxia was inversely related to the variations observed after 2 h of hyperoxia. In other words, those patients who did not demonstrate hyperoxia-induced vasoconstriction tended to show an unaltered or even decreased vessel density at return to baseline FiO_2_. Several factors including baseline PaO_2_ and ROS levels, factors affecting vascular tone, or an underlying microvascular/endothelial dysfunction may determine this “non-physiological” response. In healthy volunteers normobaric hyperoxia induced a reduction in the estimated muscle O_2_ consumption with slower StO_2_ drop during the VOT [10]. In our study, the StO_2_ downslope was unaltered after 2 h of hyperoxia, suggesting no apparent effect on muscle O_2_ consumption, which seems in contrast to the increased ScvO_2_. It should be taken into account that the response of the peripheral skeletal muscle circulation may not always reflect the response of other organs in the critically ill. Microvascular mechanisms of regulation and adaptation may vary between different capillary beds and may be impaired in a heterogeneous manner during critical illness. Sedation or vasopressors in ICU patients may influence tissue O_2_ requirements and extraction rate in the peripheral skeletal muscle circulation. Differences in the study protocol and time of exposure to hyperoxia may also contribute to the discrepant results.

Our study has several limitations. First, the non-randomized, non-blinded design of the study, together with the relatively small sample size, precludes drawing definitive conclusions. Second, we studied a necessarily heterogenous critically ill patient population, although we tried to standardize both the patients and their status (e.g. cardiorespiratory stability) as much as possible. Third, baseline differences in Hb between the two groups may be responsible for different changes in EPO levels, irrespective of the exposure to hyperoxia. In addition, we cannot exclude that differences in other parameters between the two groups (e.g. lower body temperature in the hyperoxia group) influenced microvascular perfusion and the changes detected. Fourth, the observed increase in EPO levels may not have been sufficient to induce a significant increase in erythropoiesis. The 48-hour observational period may have been too short to detect an increase in reticulocyte count and Hb after the exposure to hyperoxia. We focussed upon EPO production as numerous confounding factors may have influenced Hb levels over this time period including hemodilution due to fluid infusion, anemia of inflammation, repeated blood sampling, and other blood losses. Fifth, NO, ROS and GSH were measured only in 60% of the study population. Lastly, cardiac output and systemic vascular resistance were not measured in this stable population. As MAP was stable over the course of our study despite the local vasoconstrictor response, we speculate that cardiac output was reduced after 2 h of hyperoxia.

## Conclusions

A two-hour exposure to normobaric hyperoxia was associated with a slight increase in serum EPO levels within 48 h in critically ill patients. Mechanisms responsible for the stimulation of erythropoiesis may include an enhanced production of GSH induced by an increase in ROS. This enhanced oxidative stress was followed by normalization 2 h after returning to baseline levels of FiO_2_. An early vasoconstrictor response was observed at the microcirculatory level under hyperoxia. After 2 h , the response was heterogeneous between different patients, but normalized on returning to the baseline FiO_2_.

The findings of this pilot study encourage further exploration of the NOP as a means of stimulating erythropoiesis during critical illness. However, adequately controlled studies are needed to isolate the effect of short-term hyperoxia on EPO production independently of possible confounding factors such as baseline Hb levels or the patient’s underlying disease. Potential dangers of excessive O_2_ exposure must also be considered, specifically enhanced oxidative stress and short-term alterations in microvascular perfusion. Further investigations should clarify the time course of the microvascular response and recovery under hyperoxia in critically ill patients and identify any factors potentially influencing this response.

## Abbreviations

ANOVA: Analysis of variance
AUC: Area under the curve
BFV: Blood flow velocity
EPO: Erythropoietin
FHI: Flow heterogeneity index
FiO_2_: Inspired oxygen fraction
GSH: Glutathione (reduced form)
GSSG: Glutathione (oxidized form)
Hb: Hemoglobin
HIF-1α: Hypoxia-inducible factor-1 alpha
HR: Heart rate
ICU: Intensive care unit
MAP: Mean arterial pressure
MFI: Microvascular flow index
NIRS: Near-infrared spectroscopy
NO: Nitric oxide
NOP: Normobaric oxygen paradox
O_2_: Oxygen
ONOO: Peroxynitrite
PPV: Proportion of perfused vessels
PVD: Perfused vessel density
ROS: Reactive oxygen species
SDF: Sidestream dark field
StO_2_: Tissue oxygen saturation
THI: Tissue hemoglobin index
TVD: Total vessel density
